# Pathology of Traumatic Tetanus

**Published:** 1829-01-01

**Authors:** 


### LXVI.

### HOPITAL DU MANS.

### TRAUMATIC TETANUS—PATHOLOGY OF.

We are inclined to think, that if ever the true pathology of traumatic tetanus be discovered, it will be found, that the nerves leading from the injured part will exhibit symptoms of inflammation, and that this inflammation will extend to the spinal marrow. In a former Number* we alluded to some researches made by M. Lepelletier, surgeon of the HÔPITAL DU MANS, on the subject of tetanus traumaticus. He had scarcely published his paper, when the following interesting case occurred, confirmatory of this gentleman's doctrines.

*Case.* Peter Crepon, aged 15 years, feeble in constitution, and miserable in circumstances, had been affected with a tumour on the *left* heel, from the age of three years, which, gradually increasing, ulcerated, assumed a malignant character, and, finally led to amputation, at the Hospital of Mans, on the 28th of Dec. 1827. Nothing particular occurred till the 5th Jan. 1828, when he complained of convulsive twitchings in the stump. On the 6th, symptoms of tetanus came on, and were soon unequivocal. It was remarked, that the temporal and masseter muscles of the *left* side only were affected with trismus. This corresponded with the amputated limb. We shall not detail the daily treatment and symptoms. Repeated venesections were performed, and, in almost every instance, they relieved the tetanus for a time; but nothing could arrest the progress of the disease, and death took place on the 9th January.

*Dissection.* A small depôt of purulent matter was found in the stump, on the head of the fibula, with traces of inflammation in the neighbourhood. The sci-

---

* See No. XVI. April, 1828, p. 439.

atic nerve at this place was found so much injected as to be of a violet hue, while the same nerve, in the other limb, was of its natural colour. This discoloration continued, with some interruptions, up to the hip. The patient, during life, complained of pain in the track of this nerve, when pressed by the hand—and that for some days previous to the development of the trismus. The discoloration could be traced in some branches of the sciatic nerve distributed to the crural muscles. The pulpy substance of the nerve was found to be somewhat softened. The pia mater of the spinal marrow was decidedly injected; especially towards the middle of the dorsal region, where the medulla itself was softened and degenerated in structure. Some reddish effusion was also found under the membranes of the medulla spinalis.—Journal de Progres.

The narrator thinks that the evidence of the inflammation of nerve from the stump to the spine, as the cause of the tetanus, is here complete. We do not think that the deduction can be denied, as far as this case is concerned; but, unfortunately, there are other cases of traumatic tetanus, where the most careful dissection cannot detect any inflammation of nerve between the seat of injury and the encephalon. A case occurred at St. George's Hospital, where the spinal marrow, and the nerves of the limb were carefully examined, but without success. These conflicting facts prove that tetanus may be induced by the local injury, with or *without* evident change in the spinal marrow or nerves.

---

